# Vibration-Rotation Structure in Absorption Bands for the Calibration of Spectrometers From 2 to 16 Microns^1^

**DOI:** 10.6028/jres.064A.004

**Published:** 1960-02-01

**Authors:** Earle K. Plyler, Alfred Danti, L. R. Blaine, E. D. Tidwell

## Abstract

Suitable bands of common gases have been tabulated and remeasured wherever necessary from 2 to 16 microns to obtain an accuracy of about 0.03 cm^−1^ throughout the region and to provide good calibrating points at frequent intervals. Some 600 rotation-vibration lines are illustrated in 20 spectrograms and wavenumbers are listed in companion tables with considerable intercomparison with worthy data obtained in other laboratories. The absorpotion bands were remeasured or calibrated by using either a precisely graduated grating circle or standard atomic lines with the fringe system formed by a Fabry-Perot interferometer. Characteristic features of the individual bands are discussed briefly and references to other publications are given. The substances used for calibration include H_2_O, CO_2_, CO, HCl, HBr, NH_3_, C_2_H_2_, CH_4_, N_2_O, and polystyrene film.

## 1. Introduction

In recent years, infrared spectroscopists have expressed concern regarding satisfactory methods and wavelengths for calibrating good prism and small grating spectrometers [1, 2, 3, 4].^3^ In the past, a number of publications [5, 6, 7, 8, 9, 10, 11] from several laboratories did tend to ease the immediate requirements; but with the influx of finer instruments the need for more thorough, suitable, and precise coverage has arisen. Professor Mizushima and fellow workers have clearly indicated in their detailed measurements [12] that the small grating instruments are certainly capable of producing rather precise data. The present work addressed itself to the need for bringing together and remeasuring suitable bands of common gases in order to certify absolute accuracy to within several hundredths of a cm^−1^ throughout the region and to provide calibration points at frequent intervals.

## 2. Utility of the Molecular Band Method

Although in several cases (extremely precise measurements on CO [13, 14, 15] and HCN [16]) the use of molecular bands for calibration purposes is probably as good as the use of atomic lines, this method should not take preference over the use of atomic lines when precise measurement is being considered. However, when measurements good to several hundredths of a cm^−1^ are sufficient or in cases where other limiting factors enter, viz, broad bands, then this method may offer a number of advantages. In the case of prism instruments, the use of appropriate atomic lines for calibration is rather limited to the visible and near infrared regions and the need for single order coverage throughout is apparent. Present day commercial instruments are more easily calibrated in absorption than in emission and the molecular band method obviates the need for a full battery of atomic line equipment as well as personnel capable of using it. In addition, only available or easily obtainable substances have been used in this work so that laboratories should experience little difficulty in using this method. Most of the gases are available in typical chemistry laboratories and it will be mentioned that work in this laboratory has indicated that, in general, natural gas from commercial lines and acetylene drawn from the welder’s tank are suitable for calibration purposes. Should impurity peaks arise in these bands, use can still be made of the lines which are clearly those tabulated and illustrated in the following sections.

Some 600 lines, which provide rather thorough coverage, are tabulated and illustrated in 20 spectrograms. For certain bands, the number can be appropriately increased by having reference to recommended publications given in the discussion section. Attempts were made to keep the number of different gases used at a minimum and they include: H_2_O, CO_2_, CO, HCl, HBr, NH_3_, C_2_H_2_, CH_4_, and N_2_O. Also a polystyrene film has been included.

## 3. Instruments and Methods Used in Calibrating the Standard Lines

Two instruments in the Radiometry Section of the NBS were used in measuring the bands and in the 2- to 4-*μ* region most bands were measured on both instruments at different resolution and the results compared.

### 3.1. 5 to 16 Micron Region

#### a. Instrument

The previously described [17] grating spectrometer with an off-axis collimating mirror of 1-m focal length was used in conjunction with an extremely precise grating circle reported to be accurate to at least 3 sec of arc for any angle [18]. For the longer wave work (5 to 16 *μ*) the lead telluride detector was replaced with a thermocouple detector. When this instrument was used in the 2- to 4-*μ* region, the thermocouple detector was purposefully used so that resolution could be lowered and made comparable to that of small grating instruments. A KBr foreprism arrangement with fixed foreprism slits and a manually adjustable prism was used for order separation throughout this work. Under these stated conditions the instrument was capable of resolving lines separated by 0.3 cm^−1^ in the 7- to 16-*μ* region and up to about 1 cm^−1^ in the 2.5- to 6.5-*μ* region.

#### b. Method of Calibration

With a microscope attachment, angles were read off the inscribed circle, centered with respect to the spindle of the cone which rotates the grating, and fiducial marks recorded on the chart. During measuring runs, readings were taken every 5 min of arc and a slow drive speed (~23 min per deg) was used. It was found that proper positioning of the foreprism energy upon the entrance slit of the spectrometer proper was necessary in the sense that for extreme settings, where the maximum of energy being admitted to the spectrometer would come about 5 grating degrees away from the point of interest, the frequencies of measured lines would be in error by several hundredths of a cm^−1^. By making frequent settings of the foreprism (about every 2 deg of grating angle) so that the energy would be maximized and hence placed symmetrically on the entrance slit, these effects could be ignored. Such considerations were, of course, of greater importance in regions where wide spectrometer slits were required or at long wavelengths where the dispersion of KBr is greatest. (This matter was considered in some detail, since it is felt that it is a frequently overlooked source of error occurring with the use of fore-prisms.) In the course of any complete run (standards plus spectrum to be measured) no changes were made in instrumental conditions (viz, retardation resulting from changes in amplifier response settings may shift the line center) unless absolutely necessary.

At the start of the work, it was decided that the precisely measured CO fundamental lines (see ref. [13]) (table 4) be used as standards wherever possible. The procedure devised was that of using the simplified grating relation, *nλ=K* sin θ, where an “effective” grating constant, *K*_air_, incorporating spectrometer characteristics, could be calculated by measuring about 15 CO lines and the physical central image. This was done with the grating being turned first in one direction then in the reverse direction. If slightly different, the *K*’s obtained separately for each direction would be averaged. Once a good value of *K* had been determined for a grating, the central image was always calculated from the 10 (or so) standard lines recorded either before or immediately following a measuring run. Using this procedure, the frequency of standard CO lines could always be recalculated from chart measurements to within about ±0.01 cm^−1^ of the accepted value. With good spectrometer and grating alinement, it was felt that CO standards could be used to calibrate NH_3_, CH_4_, and H_2_O lines even though the grating angle for the standards differed appreciably from that of the lines in question. In calibrating the lines, both standards and runs to be measured would be recorded with the grating being turned in the same direction. As is often the case, it was found that slightly different values would be obtained for the two directions. Final values represented the average of an equal number of measurements in each direction. It is believed that the tabulated values are accurate to ±0.02 cm^−1^ in this region.

#### c. Chart Reduction

Wherever possible single lines were picked out for measuring, but in some instances certain complex lines were measured since they are particularly suited for direct prism observation. It is advisable to record spectra on ruled chart paper where the ruled lines are reproducible perpendicular to the chart edge. The determined center point of the line to be measured can then be easily projected to a suitable measuring base line on the chart by using a pair of dividers and the nearest ruled line. The center of the line should be determined with a pair of stiff dividers at about the 2/3 absorption point and not at the peak or apparent maximum of absorption. If the spectrum is particularly noisy the center should be determined at a number of places from the 2/3 to 3/4 point of the maximum of absorption and the average taken. Fractional distances between “pip” marks should be measured to within 0.005 in. using a steel rule and visual enlarger if necessary. For the majority of measuring runs, 5 min of grating are corresponded to 2 in. on the chart.

#### d. Instrument and Calibration

The instrument, which uses the double-pass system and is capable of resolving about 0.03 cm^−1^ has been described [19] and only a few details need be added. Standard atomic lines and the fringe system described in a previous paper (see ref. [17]) were used to calibrate the molecular bands. By scanning the spectrum slowly and averaging several runs, a set of relative values for the lines of a band can be determined to a few thousandths of a cm^−1^ and the absolute to ±0.01 cm^−1^. With scanning at a medium rate, absolute values are probably in error by ±0.03 cm^−1^. The accuracy of measurement of this instrument is increased by the lines being very narrow (0.03 cm^−1^). Measurements made under lower resolution (about 1 cm^−1^) with the other instrument in this region agreed with these to about ±0.05 cm^−1^. This indicates that low resolution spectrometers can measure with an error of about 1/10 of the resolution.

#### e. Refractive Index

In this laboratory measurements on both instruments are carried out with the grating in air so that reported frequencies were corrected to vacuum by using refractive index tables, and temperature and pressure corrections given by Penndorf [20].

## 4. Comments on Measuring Technique and Use of the Standard Lines

For the present-day molecular spectroscopist who wishes to carry out precise measurements, useful points on measurement technique, whether on standard or unknown lines, have already been mentioned in previous sections on method of calibration, instrument performance, chart reduction and also in reference [17]. Concerning a spectrum to be measured, in cases where the instrument is being used at or near its resolution limit (ripple spectrum) or in the case of complex lines or shoulders, then the best possible “center” point must be picked and this may necessarily have to be near the absorption peak since lines may not be well enough developed to permit measurement at the 2/3 to 3/4 absorption point.

It is advisable to record both spectra and standards for each direction of grating turn if the driving mechanism is equally accurate in both directions. Provisions or modifications should be made on commercial or laboratory instruments so that the grating can be used in both directions with near equal accuracy and the results averaged. Undoubtedly, in most laboratories the standards will be used in conjunction with reproducible pip marks. Data will be extracted from simple pip or drum number versus frequency (cm^−1^) relations by fitting a straight line to two end points and an extended deviation curve plotted. In view of extremely precise data for certain bands (CO, HCN) the utility of using these standard lines in higher orders for the same grating angle should not be overlooked. In terms of precision measurement, it would appear that the *R* branch (1,000 to 1,200 cm^−1^) of the *μ*/_2_ NH_3_ band might also be classified in this category. This higher order procedure, of course, requires refractive index considerations for conversion to vacuum and each spectral region must be handled independently.

In making use of the standard lines under lower or higher resolution, workers should exercise necessary care. For example, in the CO bands where (apart from some isotopic overlap) the lines are single and suitably spaced throughout, even ripple peaks observed with a prism instrument would serve as satisfactory calibration points. Whereas, in many other bands proper usage under lower resolution may require judicious use of sufficiently low pressures and slow scanning speeds to assure the proper development of lines and eliminate “pulling” by strong neighboring absorption.

## 5. Discussion of Spectograms

In general, the runs made on white chart paper for illustration purposes were made at somewhat faster speeds and with much more chart concentration than was the case for the measuring runs. On some of the illustrations, sloping or bowing backgrounds are due either to foreprism effects or the joining of separate sections.

### 5.1. CO_2_, *v*_2_ Fundamental

**Figure 1:**
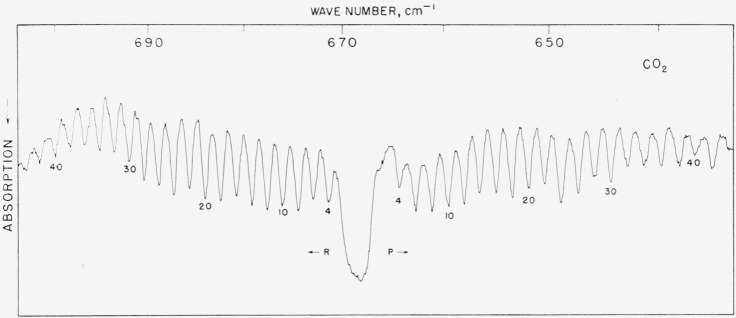
The v_2_ fundamental of CO_2_ recorded in the first order of an 1,800 lines/in. grating under atmospheric conditions and 7-m path Spectral slit width about 0.5 cm^−1^.

Some 20 lines in this band were remeasured and comparison is made with the work of Rossman, Rao, and Nielsen [21–A, 21–B] and also with the values reported by Mizushima et al. (see ref. [12]). Although our measurements were somewhat handicapped by the overly strong absorption (7-m path) and also by poorer grating resolution in this region, the agreement is quite good. It appears that the values reported by Rossman, Rao, and Nielsen (also additional unpublished work communicated by Dr. Rao) are accurate and that lines they report both at higher and lower frequency as well as those given in table 1 may be used for calibration purposes provided the atmospheric absorption is augmented by CO_2_ used in a vapor cell. The spectrum is illustrated in figure 1.

### 5.2. Acetylene, *v*_5_ Fundamental

**Figure 2:**
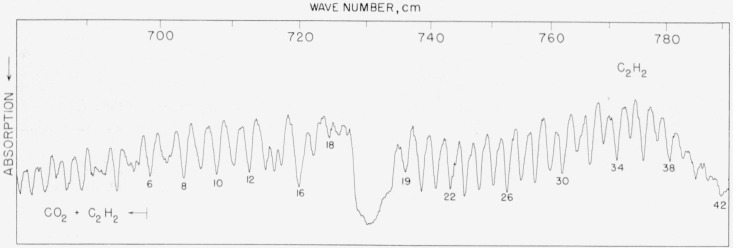
The v_5_ fundamental of acetylene recorded in the first order of an 1,800 lines/in. grating with 8-cm pressure and 5-cm path Spectral slit width about 0.7 cm^−1^. The low frequency end of the *P* branch overlaps with atmospheric absorption of CO_2_ and the strong *Q* branch (hot band) of CO_2_ at 720.46 cm^−1^ also contributes to absorption at this point.

This band was included in order to provide useful calibration points between the end of the strong 15-*μ* CO_2_ band and the ammonia band. On the low-frequency end, our values are in excellent agreement with the recently published values of Rao, Ryan, and Nielsen [22] and table 2 includes additional values as reported by these authors. At higher frequency, results are also compared with the work of Christensen, Eaton, Green, and Thompson [23]. The agreement is quite good except between lines 29 to 39, where several lines appear to be pulled out of position by an overlapping band. These lines are set out by parentheses. Results of recent measurements by Jones and Nadeau of the National Research Council, Ottawa, Canada are also included. The spectrogram is illustrated in figure 2.

### 5.3. NH_3_, *v*_2_ Fundamental

**Figure 3:**
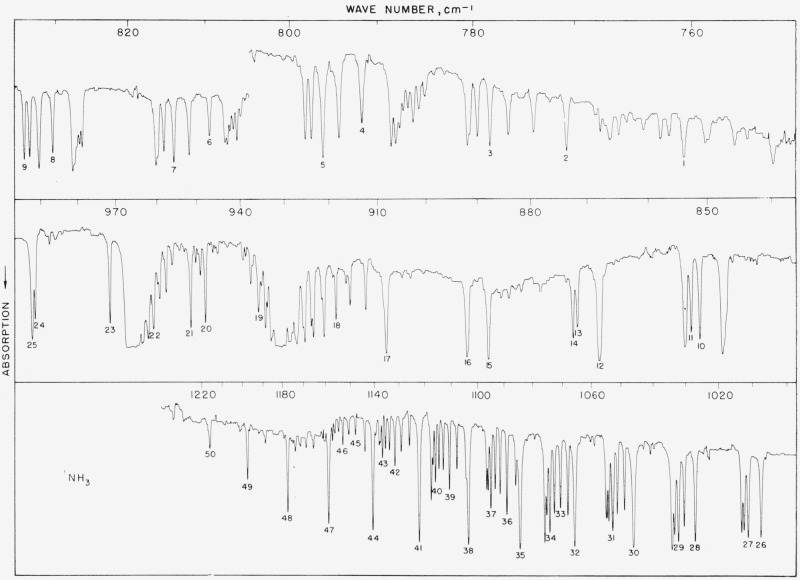
The v_2_ fundamental of *NH_3_* recorded in the first order of a 3,600 lines/in. grating with 7-cm pressure near the band center and 12-cm for the wings, both with 5-cm path. Spectral slit width about 0.3 cm^−1^.

Table 3 gives values obtained in four different laboratories for this band. Since these four works are of about equal quality, the average value reported in the last column represents a simple numerical average (within arbitrary ±0.04-cm^−1^ exclusion conditions). The average values are probably better than individual determinations, and it is estimated that they are good to within ±0.01-cm^−1^ absolute on the average. The region from 1,000 to 1,200 cm^−1^ is particularly good. We have cause for suspicioning some of our low-frequency lines (numbers 1, 3, and 6) due to the fact that the grating was being used at exceedingly high angle. As more experimental and theoretical data become available for ammonia, a good set of calculated values may ultimately replace the average value column. The spectrum is shown in figure 3.

### 5.4. Methane, *v*_4_ Fundamental

**Figure 4:**
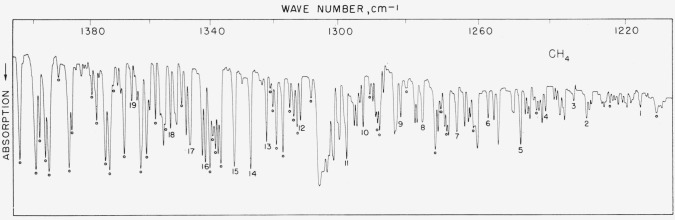
The v_2_ fundamental of *CH*_4_ recorded in the first order of a 3,600 lines/in. grating with 12-cm pressure and 5-cm path Spectral slit width about 0.5 cm^−1^. The open circles refer to atmospheric water peaks.

This band serves the purpose of providing connection between the ammonia and water bands and is included mainly because workers with small instruments may have difficulty in observing weak water lines in this region. We are unable to compare these numbers with others, as there are no other precision measurements available but our results (see table 4) would appear to be good to about ±0.02-cm^−1^ absolute. The spectrum is shown in figure 4.

### 5.5. H_2_O, *v*_2_ Fundamental

**Figure 5:**
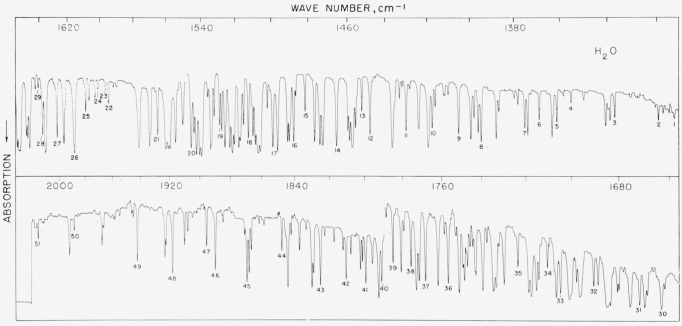
The v_2_ fundamental of *H*_2_*O* recorded with a 3,600 lines/in. grating under atmospheric conditions (T=21° C, humidity 21%) and 7-m path The upper spectrogram was recorded in the first order and the lower in the second order. Spectral slit width about 0.6 to 0.7 cm^−1^ for both spectrograms.

Some 50 good lines of varying intensity were picked out for measurement. (See table 5.) Wherever possible, the lines are compared with recent precision determinations of Rao, Ryan, and Nielsen (see ref. [22]). These authors indicate that their values are, on the average, about 0.07-cm^−1^ lower than those reported by Dalby and Nielsen [25] for the region 1,450 to 1,650 cm^−1^. Our results, when compared with Dalby and Nielsen’s values, would indicate that this is the case throughout the band. Wherever possible, mild flushing or pumping down of the spectrometer housing is to be recommended for cleaner development of the lines but our results indicate that good calibration measurements can be made even on saturated lines provided the line is not a complex one. This band is illustrated in figure 5.

### 5.6. CO Fundamental

**Figure 6:**
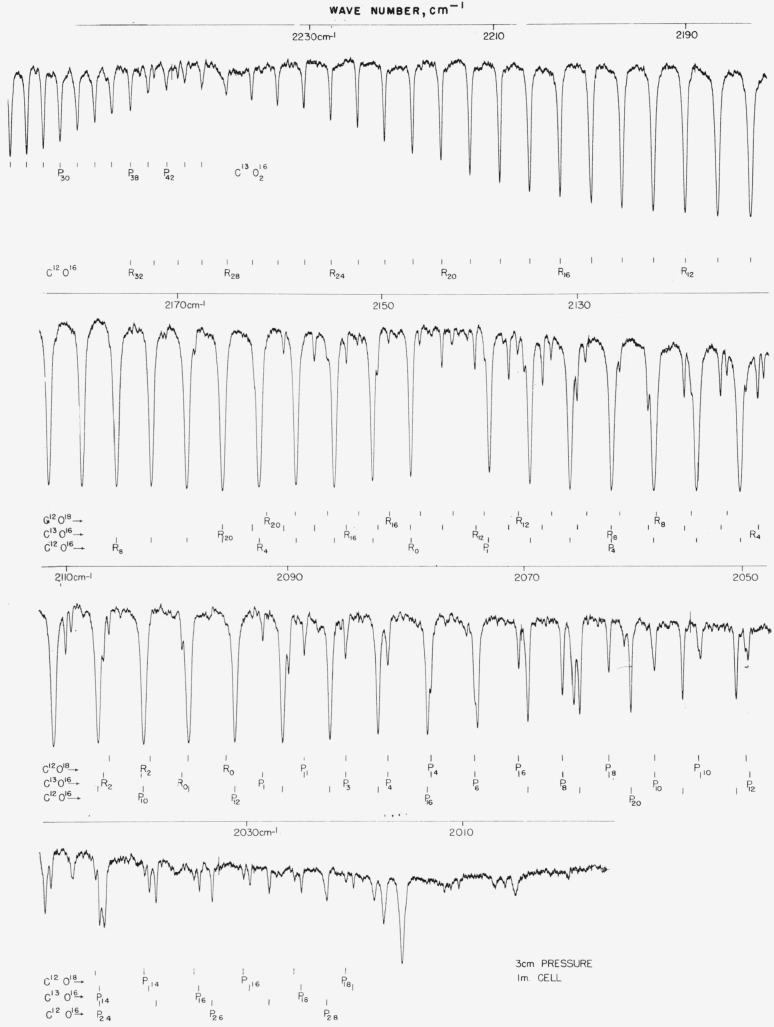
Absorption band of *CO* recorded in the first order of a 7,500 lines/in. grating with 3-cm pressure and 1-m path Spectral slit width about 0.3 cm^−1^.

The CO fundamental band at 4.67 *μ* was measured accurately by Plyler, Blaine, and Connor (see ref. [13]) in 1955. With the measurement of the CO harmonic (2.33 *μ*), it was possible to calculate the position of the fundamental band. This has been done by Rank and his colleagues (see ref. [14]) and a very good agreement with the observed values was obtained. There was a slight difference in values for the high *J*’s in the *R* branch. Recently the *R* branch has been remeasured and a better correspondence with the calculated values is obtained. The accuracy of the calculated values approaches a few thousandths of a cm^−1^. The spectrum is shown in figure 6 and the values are listed in table 6.

### 5.7. C^13^O_2_, *v*_3_ Fundamental

The C^13^O_2_ band is excellent for calibration from 2,240 to 2,280 cm^−1^ and connects with the *v*_3_ band of C^12^O_2_. This spectrum was measured in 1955 by Plyler, Blaine, and Tidwell [26]. In this work, the lines of this band have been remeasured and the values check closely with the first determination. Unfortunately, the *J* values were incorrectly listed in table 1 of the above publication and each *J* of the *P* branch should be reduced by 2; that is, *P* 44 should be *P* 42, etc. The work with a small grating spectrometer of Mizushima and his colleagues (see ref. [12]) has also been included and there is very good agreement between the two lists of frequencies. The spectrum of this band is shown in figure 7–A and the values are listed in table 7–A.

### 5.8. C^12^O_2_, *v*_3_ Band

The only precision work for this band is the work which was done at the NBS. The frequencies were first published in 1955 (see ref. [26]) and since then two other determinations have been made. With the long paths employed in this laboratory, it is not possible to measure the central part of the band. Figure 7–B shows how use can be made of the entire band by purging the instrument housing and containing the CO_2_ in a cell. (Dr. Norman Jones of the National Research Council of Canada has furnished the spectogram as observed with a purged small grating instrument.) Table 7–B gives observed values for the band wings and calculated values for the central part. In view of the fact that molecular constants calculated from the observed lines agree well with those of Courtoy [27] obtained from many bands and agreement with observed lines is good, it is felt that workers can use either the observed or calculated values of table 7–B equally well provided the intensity and resolution of the band is close to that of figure 7–B. Both observed and calculated values should be good to about ±0.02-cm^−1^ absolute. Since the band consists of a collection of no less than four overlapped bands, results are subject to being extremely resolution sensitive and workers should not use lines exhibiting pronounced overlap. (The *R* branch is comparatively free of such difficulties.)

### 5.9. HBr Fundamental

As shown in figures 8–A and 8–B, this band was measured under considerably different resolution. With the conditions describing figure 8–A, there was no evidence to the effect that the components arising from HBr^79^ and HBr^81^ were being resolved out. This was done intentionally to see whether, when measured as a single peak, the frequency agreed with the average determined for each component under resolution approaching 0.05 cm^−1^. The low resolution single-peak frequency agreed with the high resolution average value to within 0.05 cm^−1^ on the average (See table 8). This relatively good agreement does indicate that the high resolution results are correct in the absolute sense and that apart from greater inability to determine the center of the single peak (near 1-cm^−1^ half-width), its frequency is given well enough by the component average. In table 8, the high resolution average of components is the recommended value for single peak use. Calculated values were also determined for the high resolution results and the values given in the first column of table 8 are believed to be accurate to within ±0.02-cm^−1^ absolute.

### 5.10. HCl Fundamental

**Figure 9:**
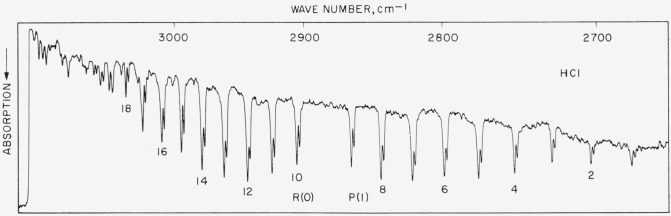
Absorption band of *HCl* recorded in the second order of a 5,000 lines/in. grating with 20-cm pressure and 5-cm poth Spectral slit width about 1.0 cm^−1^. Weak atmospheric water lines of the 2*v*_2_ band begin to appear at the high frequency end.

The spectrum illustrated in figure 9 was recorded under low resolution and the entire band shown in this way for the sake of compression. Figure 8–B shows 3 lines of the band under high resolution. The band was measured on both instruments in this laboratory and results are compared with values reported by Mills, Thompson, and Williams [28] (see table 9). Molecular constants were obtained from the high resolution observations and calculated line frequencies agree well with the observed. In general, the high resolution results of this work fall between the low resolution values and those of Mills, Thompson, and Williams. The high resolution work does not, however, represent precision measurement, since the lines were measured at medium-fast scan in order to appropriately cover this extremely wide band in reasonable time. The observed values given in the table for the high resolution work are to be preferred and represent the best possible set. These values should be good to about ±0.02-cm^−1^ absolute. (Future precision measurements are contemplated for this band.)

### 5.11. Methane, *v*_3_ Band

**Figure 10:**
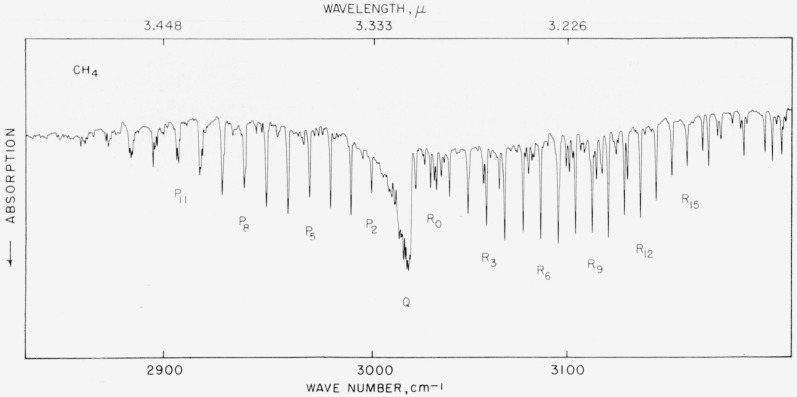
The v_2_ fundamental of methane recorded with a 7,500 lines/in. grating with 10-cm pressure and 5-cm path. Spectral slit width about 0.9 cm^−1^, certain weak lines occurring between methane lines are part of the 2*v*_2_ band of atmospheric water vapor.

The *R* branch of this band and some of the lower-*J* lines of the *P* branch are fairly suitable for providing calibration points in this region. However, care should be exercised to the extent of staying within the bounds of the resolution illustrated in figure 10. Under higher resolution the lines begin to break up, *P* 4, for example, shows four components within 0.37 cm^−1^ and frequency allocation becomes difficult. The values are listed in table 10 and strongest components should be used where indicated.

### 5.12. Acetylene Bands Near 3,300 cm^−1^

**Figure 11:**
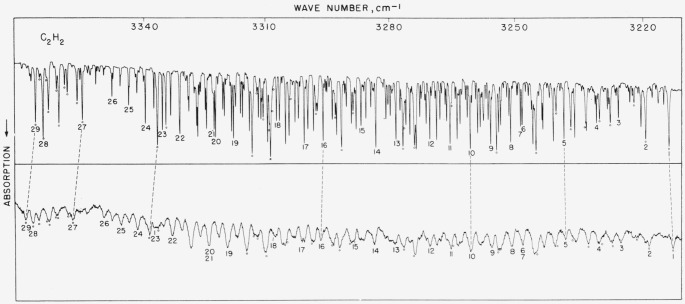
The two absorption bands of acetylene near 3,300 cm^−1^ compared under high and lower resolution The upper spectrogram was recorded with a 10,000 lines/in. grating singly passed with 1.5-cm pressure and 5-cm path. The lower spectrogram was recorded in the second order of a 5,000 lines/in. grating with 4-cm pressure and 5-cm path. Spectral slit widths about 0.10 and 0.8 cm^−1^, respectively. Atmospheric water lines of the 2*v*_2_ band are denoted by open circles.

The spectrogram of figure 11 gives a rather interesting comparison of lines and peaks under considerably differing resolution. The scale on the lower panel, though close to that of the upper one differs somewhat and is indicated by the tie lines. The values listed in table 11 indicate the rather remarkable feature that even under considerably lower resolution single or symmetrical lines can still be accurately measured. The high resolution measurements were made with fringes and the grating circle was used for the lower resolution measurements. In two cases (lines 6 and 7; 20 and 21), two closely spaced and nearly equal intensity components were measured as a single peak and this value compared with the average of the two components. As in the case of HBr, the agreement is quite satisfactory. The results of this work are compared with values reported by Christensen, Eaton, Green, and Thompson (see ref. [23]).

### 5.13. Water and Carbon Dioxide Bands Near 3,700 cm^−1^

The spectrogram (fig. 12–A), illustrating the atmospheric absorption in this region, was recorded with the low resolution instrument so that results would approximate those attainable with small grating intruments. The indicated lines were measured under these conditions and in table 12–A the results are compared with the high resolution and high precision work of Plyler and Tidwell [29]. The agreement is satisfactory and indicates that even with lower resolution, good measurements in the order of 1 part in 100,000 can still be made. The higher resolution numbers are the recommended ones and if more frequent calibrating points are required, workers should make use of reference [29].

### 5.14. CO_2_, 021, and 101 Bands

Plyler and Tidwell (see ref. [29]) report the precise measurement of many of these lines. Certain others, which could not be measured due to overlapping water lines, are not listed in table 12–B. Most of these lines are good to about ±0.02 cm^−1^. The two bands are illustrated in figure 12–B as recorded with a nitrogen-purged small grating instrument with CO_2_ in a cell. (The spectrogram was recorded by Dr. Norman Jones, National Research Council, Ottawa, Canada.)

### 5.15. C_2_H_2_, *v*_1_+*v*_5_ Band

**Figure 13:**
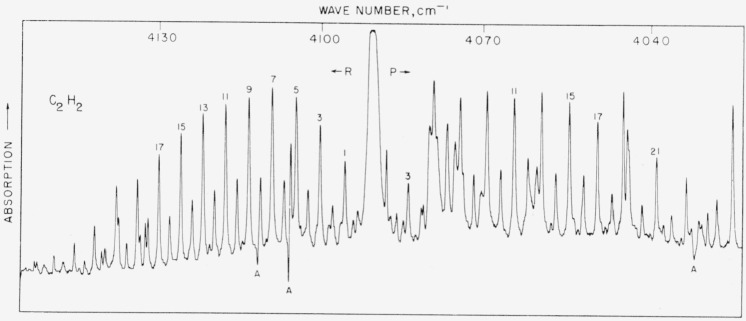
The v_1_+ v**_5_** band of acetylene recorded with a 15,000 lines/in. grating singly passed with 1-atm pressure and path Spectral slit width about 0.08 cm^−1^.

From the end of the *P* branch of the CO harmonic to the beginning of the *μ*_3_ water vapor band there is a gap of about 100 cm^−1^. There is a band of acetylene of medium intensity that falls in this region (4,039 to 4,130 cm^−1^). This band is overlapped by four weaker bands and when measured with low pressure and high resolution many lines are observed [30]. When a pressure of 1 atm is used in a 10-cm cell, the smaller bands are not observed and the *P* and *R* branches of the band stand out clearly. A number of the strong lines were measured under both high and low pressures and the values agreed closely. The numbers listed in table 13 are the results of the low pressure measurements. The spectrum is shown in figure 13.

### 5.16. CO Harmonic Band

**Figure 14:**
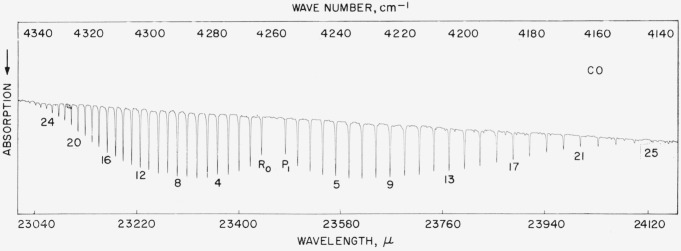
The 2–0 band of *CO* recorded with a 15,000 lines/in. grating with 20-cm pressure and 60-cm path Spectral slit width about 0.15 cm^−1^.

The CO harmonic band at 2.34 *μ* has been measured very accurately by Rank and his colleagues (see ref. [14]) and also by Plyler, Allen, and Tidwell (see ref. [15]). This band is one of the few which have been measured in the infrared to such a precision that the reported frequencies are accurate to a few thousandths of a cm^−1^. This spectrum was measured with a 60-cm cell and 20-cm pressure, but a 10-cm cell with 50-cm pressure is sufficient for observing forty lines of this band. The spectrum is shown in figure 14 and the frequencies are listed in table 14.

### 5.17. N_2_O, 2*v*_1_ Band and Hot Band

**Figure 15:**
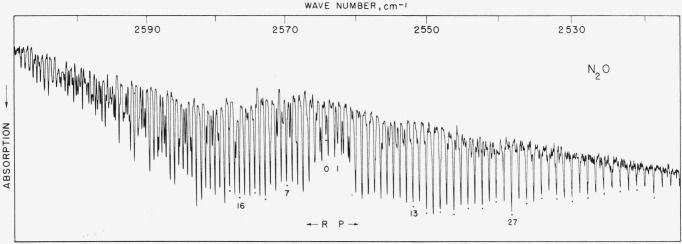
The 2v_1_ **b** and (2564 cm^−1^) of *N*_2_*O* together with the hot band (2v_1_+v_2_−v_2_, 2577 cm^−1^) recorded with a 10,000 lines/in. grating with 2-mm pressure and 6-m path Spectral slit width about 0.08 cm^−1^. The dots denote the measured and tabulated lines.

The 2*v*_1_ band of N_2_O has been measured in the region from 2,520 to 2,580 cm^−1^ and the results are shown in figure 15 and table 15. Thompson and Williams [31] had previously measured this band and their values are used to compare with the results obtained in this work. The lines which have been measured are noted by a dot on the spectrogram. There is an overlapping structure of weak lines and only those lines were measured which appeared in the open. This band can be resolved by a small grating instrument if a photoconducting cell is used as the detector and could be used as an alternate for HBr in this region. Also there are several regions where lines are closely grouped and the band can be used to check instrumental performance and resolution. The measurements of Thompson and Williams agree, on the average, to within ±0.04 cm^−1^ of the values in this work. There is a small shift in the direction of lower frequency of all the values of the present measurements. An average of the two sets of values would probably be correct to ±0.03 cm^−1^, which should be entirely adequate for calibrating medium resolution instruments.

### 5.18. NH_3_, *v*_4_ Fundamental

**Figure 16:**
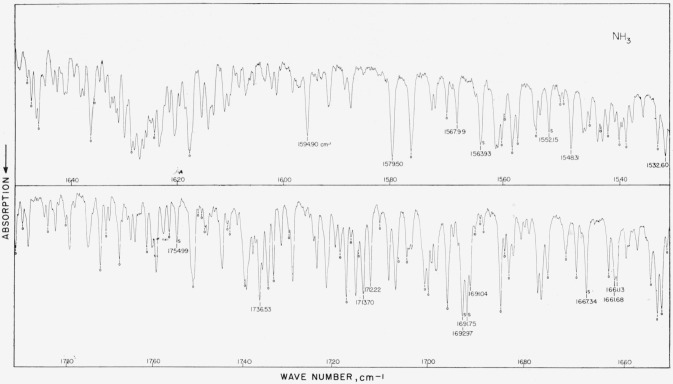
The v_4_ band of *NH*_3_ at 1628 cm^−1^ recorded with a nitrogen-purged small grating instrument Certain strong lines of the v_2_ water band still appear. A gas pressure of 7.5 cm in a 10-cm cell was used. Spectral slit width about 0.50 cm^−1^.

This band is being included as an alternate to the 6-*μ* water band and should prove of some use to workers who wish to flush their instruments or use double-beam operation to study samples contained in cells. Dr. W. S. Benedict, of the Johns Hopkins University, has picked out of the band (in accordance with the resolution illustrated in fig. 16) some 19 single or not-too-complex absorption peaks and has determined the best possible frequencies from the higher resolution results of Garing and Nielsen (see ref. [24]) and unpublished results from the National Bureau of Standards Laboratory. The results of these two works agree to within about ±0.02 cm^−1^. The frequency assigned to certain absorption peaks which appear single in figure 16 but which actually consist of several components is, of course, no longer applicable if the peak is broken up into its components under slightly higher resolution. (The letter *S* in figure 16 indicates a single line.) No table accompanies this spectrogram but instead the acceptable values which are probably good to ±0.02 cm^−1^ for single lines and off by as much as ±0.2 cm^−1^ for some complex lines, have been printed on the illustration. For calibration purposes, the *μ*_2_ water lines (see table 5) are considerably better than the lines of this band. (The spectrogram was recorded by Dr. Norman Jones, National Research Council, Ottawa, Canada.)

## 6. Polystyrene Film

**Figure 17:**
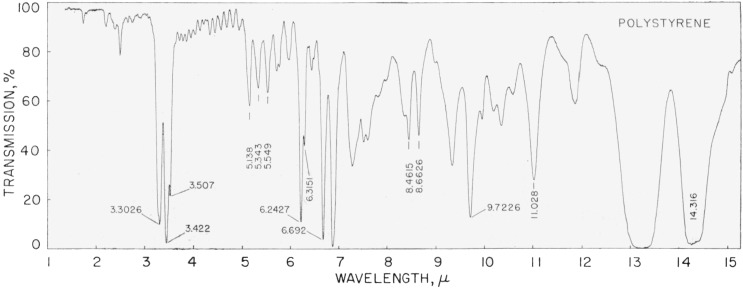
Infrared absorption spectrum, from 2 to 15 μ, of a 50-μ film of polystyrene recorded with a rock-salt prism instrument.

In 1950 Plyler and Peters (see ref. [7]) measured the infrared absorption spectrum of polystyrene on a grating instrument and certain bands were suggested for use in calibrating prism instruments. Since that time, films of polystyrene have been widely used in many laboratories and it has been found very useful in checking the calibration of an instrument in certain regions to ascertain if there are any changes in the reading of the wavelength or frequency scale of the instrument. There is a question as to the suitability of polystyrene for a calibrating material as it is not completely stable and has a different absorption spectrum when it ages. Also different batches of the material may show differences in absorption. The variation in the absorption spectrum of different films can easily be demonstrated, but the strong bands are not appreciably changed in wavelength by age or the origin of the film. In this work, we checked a film which had been made ten years ago and it showed no appreciable shifts in the absorption bands. Eight bands from 6 to 14.4 *μ* have been remeasured and the results are compared with the previous measurements of 1950 and the additional measurements of Plyler, Blaine, and Nowak (see ref. [11]) in 1957. The spectrum is shown in figure 17 and the wavelengths and wave numbers in vacuum are listed in table 16. When the faces of the film are parallel, interference fringes appear but these usually occur between 4 and 6 *μ* for a 50-*μ* film and do not change the position of the bands beyond 6*μ*. The small band at 6.3151 *μ*, which is on the long wavelength side of the strong band at 6.24 *μ*, is completely resolved by a low resolution grating instrument and is not overlapped by atmospheric water lines. It should be useful for calibration in this region. The strong band at 14.316 *μ* was measured with a film of 8-*μ* thickness and with this film the maximum of absorption could be accurately determined. It is estimated that the bands listed in table 16 are accurate to ±0.3cm^−1^ and should be useful in calibrating low resolution instruments, but should not be considered as being of the same order of accuracy as the molecular vapor bands. In measuring the polystyrene bands, the center was determined near the ¾-absorption point for symmetrical bands and reference should be made to this point when making use of the numbers in table 16.

## 7. Summary

The rather “popular” ammonia band at 3 *μ* was not used in this work because of its resolution sensitiveness and also because of temperature and pressure effects. For example, there are 12 components in the *P* 8 “line” extending over 1.5 cm^−1^. Under low resolution (see ref. [11]) the *Q* branch, which extends over 5.5 cm^−1^ and consists of hundreds of lines is probably good to 0.5 cm^−1^.

The 16-*μ* coverage may be appropriately extended by making use of frequency values reported by Lakshmi, Rao, and Nielsen [32] for N_2_O. In this work, we have checked Rao’s measurements on the 15-*μ* CO_2_ band and feel that if the same techniques were used on the N_2_O measurements, then these numbers may be used. Considering the lower resolution used by the Japanese workers (see ref. [12]) on this band, the agreement with values reported in [32] above is quite good.

At the high frequency end, we have already given reference to the work of Rank and coworkers on HCN (see ref. [16]) and wish to include also the grating work on H_2_O vapor at 1.9 *μ* and on CH_4_ at 2.2 *μ* as given in reference [8].

The calibrating values given in this report should make it easier for infrared spectroscopists to obtain accurate measurements in the region from 2 to 16 *μ.* As further measurements are carried out in other laboratories, there will be more values for intercomparison and it should be possible to obtain calculated values for certain bands which would be more accurate than the observed values from any one laboratory. No recommended frequencies representing properly weighted averages of the work of several laboratories have been given, but this may be done at a later date when more data are available. It is hoped that spectroscopists will send new data of this type to the authors for wavelength standards so that it may be incorporated in a future report. It is also requested that any corrections or suggested changes be communicated to the authors.

## Figures and Tables

**Figure 7–A:**
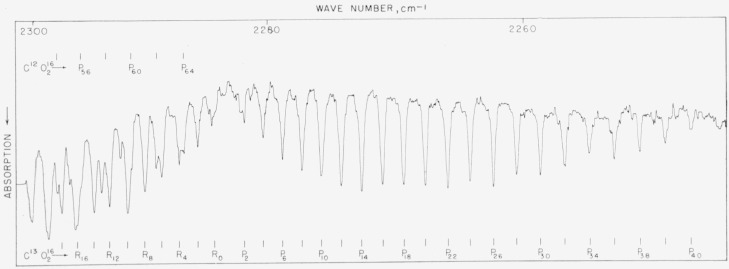
The v_3_ fundamental of *C*^13^*O*_2_^16^ recorded in the first order of a 7,500 lines/in. grating under atmospheric conditions and 6-m path Spectral slit width about 0.3 cm^−1^.

**Figure 7–B:**
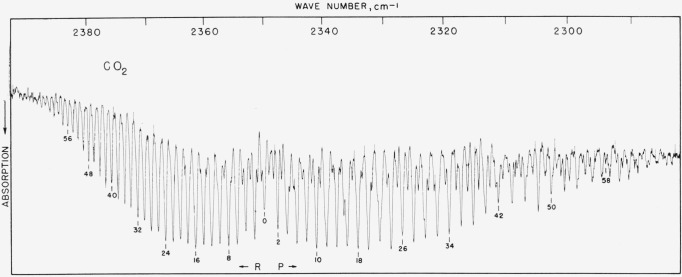
The v_3_ fundamental of *C*^12^*O*_2_^16^ recorded in the third order of a 70 lines/mm grating with a small grating instrument The spectrometer housing was purged of atmospheric CO_2_ with dry nitrogen and the spectrum recorded with 30-mm CO_2_ in a 10-cm cell. Spectral slit width about 0.25 cm^−1^.

**Figure 8–A:**
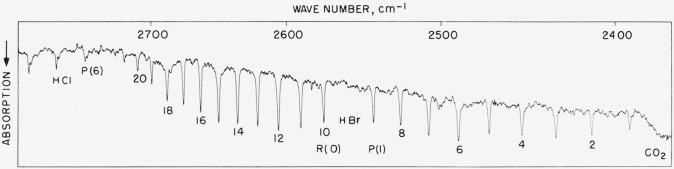
Absorption band of *HBr* recorded in the second order of a 5,000 lines/in. grating with 40-cm pressure and 5-cm path Spectral slit width about 0.8 cm^−1^. The CO_2_ band just appears at the low frequency end and the HCl band at the high frequency side. HCl was present in the HBr as an impurity.

**Figure 8–B:**
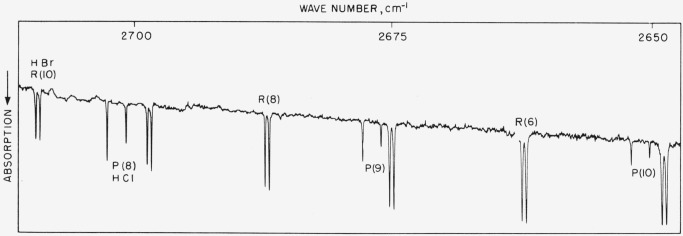
A section of the *HBr*^79,81^ bands with *HCl*^35,37^ overlapping Recorded with a 10,000 lines/in. grating singly passed with 2.5-mm pressure of each acid in a 6-m cell. Spectral slit width about 0.06 cm^−1^.

**Figure 12–A:**
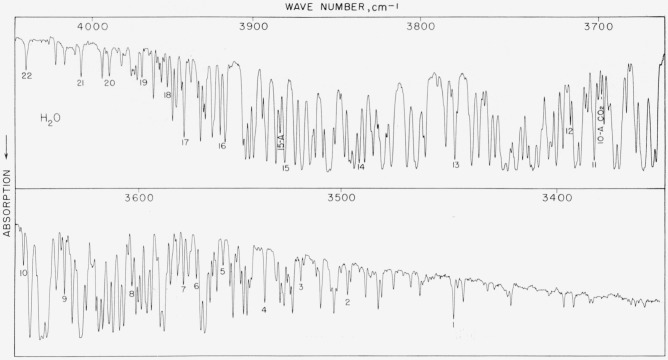
Atmospheric absorption bands of *H*_2_*O* and *CO*_2_ in the 3,700-cm^−1^ reqion recorded under intermediate resolution in the second order of a 5,000 lines/in. grating The path was 7 m with 48 percent humidity at 23° C. Spectral slit width about 0.7 cm^−1^.

**Figure 12–B:**
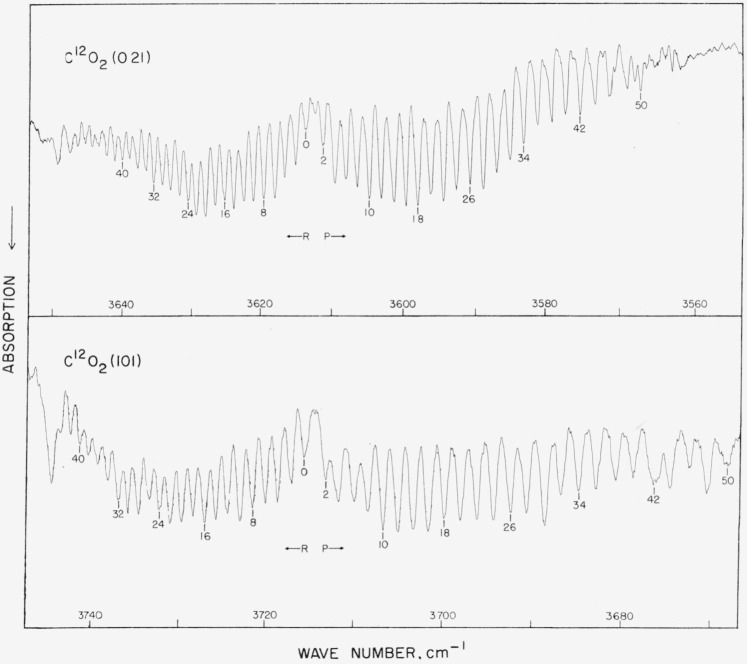
The 021 and 101 hands of *CO*_2_ recorded with a purged small grating instrument The CO_2_ was contained in a 10-cm cell at 25-cm pressure for both bands. Spectral slit width about 0.30 cm^−1^.

**Table 1:** Absorption lines of v_2_ fundamental of CO_2_ from 685 to 700 cm^−1^

*J*	*R*(*J*)			*P*(*J*)		

	Rossman, Rao, and Nielsen^a^ *v* cm^−1^ (vac.) observed	Thiswork *v* cm^−1^ (vac.) observed	Mizushima et al.^b^ *v* cm^−1^ (vac.) observed	Rossman, Rao, and Nielsen^a^ *v* cm^−1^ (vac.) observed	Thiswork *v* cm^−1^ (vac.) observed	Mizushima et al.^b^ *v* cm^−1^ (vac.) observed

2	669.75	………	………	665.83	………	………
4	71.34	………	28	64.28	………	………
6	72.88	.88	.87	62.71	………	………
8	74.45	.46	.43	61.18	.15	.15
10	76.02	.06	.02	59.64	.55	.60
12	77.60	………	.59	58.08	.06	.00
14	79.20	………	.16	56.55	.51	.53
16	80.78	………	.76	55.02	4.98	.00
18	82.36	.39	.35	53.48	.48	.47
20	83.95	.98	.95	51.95	.91	.94
22	85.55	.56	.55	50.41	.39	.41
24	87.16	.17	.14	48.90	………	.89
26	88.77	.79	.78	47.39	………	.38
28	90.38	.36	.36	45.90	.89	.87
30	91.99	.97	.99	44.37	………	.39
32	93.59	………	.60	42.86	………	.88
34	95.19	………	.20	41.35	.40	.38
36	96.82	………	.82	39.83	………	.90
38	98.44	………	.42	38.34	………	.35
40	700.07	………	.04	36.87	………	.89
42	01.69	………	.68	35.38	………	.40
Q Branch	c(10°0→01^1^0)	720.46	………			

a Rossman, Rao. and Nielsen [21].

b Mizushima et al. [12].

c The measuring point of this band was taken at the 2/3 absorption point.

**Table 2:** Absorption lines of v_5_ fundamental of acetylene from 680 to 790 cm^−1^

Line serial number	This work *v* cm^−1^ (vac.) observed	Rao, Ryan, and Nielsen^a^ *v* cm^−1^ (vac.) observed	C., E., G., and T^b^ *v* cm^−1^ (vac.) observed	Jones and Nadeau^c^ *v* cm^−1^ (vac.) observed

1	………	686.77	………	686.66
2	………	89.11	………	89.15
3	………	91.47	………	91.39
4	………	93.80	………	93.79
5	………	95.19	………	96.15
6	693.53	98.52	698.50	98.57
7	………	700.89	700.88	700.88
8	703.28	03.24	03.19	03.23
9	d(05.66)	05.60	05.56	05.53
10	07.96	07.96	07.94	07.94
11	10.30	10.30	10.37	10.29
12	12.68	………	12.67	12.60
13	15.08	………	15.03	15.12
14	16.36	………	………	………
15	17.35	………	17.35	17.35
16	19.90 (peak)	………	19.90	19.84
17	(22.15)	………	22.06	21.96
18	24.39	………	24.35	
19	36.17	………	36.18	36.14
20	38.56	………	38.56	38.54
21	40.89	………	40.88	40.85
22	43.29	………	43.31	43.20
23	45.64	………	45.64	45.65
24	47.97	………	48.02	47.95
25	50.36	………	50.37	50.29
26	52.66	………	52.74	52.70
27	(55.12)	………	55.14	55.10
28	57.42	………	57.45	57.43
29	(59.85)	………	59.86	59.68
30	62.11	………	62.18	62.10
31	(64.46)	………	64.52	64.50
32	66.74	………	66.87	66.68
33	69.08	………	69.21	69.13
34	71.39	………	71.53	71.46
35	73.71	………	73.84	73.80
36	(76.02)	………	76.17	76.12
37	78.42	………	78.52	78.42
38	(80.70)	………	80.82	80.73
39	83.07	………	83.09	83.00
40	85.43	………	85.45	85.37
41	87.75	………	87.74	………
42	90.10	………	90.12	………

a Rao, Ryan, and Nielsen [22].

b Christensen, Eaton, Green, and Thompson [23].

c Jones and Nadeau, National Research Council, Ottawa, Canada, unpublished work.

d Parentheses around values in second column indicate poorer lines.

**Table 3:** Absorption lines of v_2_ fundamental of NH_3_ from 760 to 1,210 cm^−1^

Line serial number	This work *v* cm^−1^ (vac.) observed	Garing and Nielsen^a^ *v* cm^−1^ (vac.) observed	Price and co workers^b^ *v* cm^−1^ (vac.) observed	Mimshima et al.^c^ *v* cm^−1^ (vac.) observed	Average value^d^ *v* cm^−1^ (vac.)

1	760.72	.69	.69	………	.70
2	70.96	.91	.90	.96	.93
3	78.33	.29	.27	.33	.30
4	91.76	.72	.76	.75	.75
5	96.17	.14	.16	.15	.16
6	809.76	.72	.71	.74	.73
7	14.27	.25	.22	.24	.24
8	30.68	.65	.70	.67	.68
9	34.86	.82	.84	.83	.84
10	51.36	.32	.36	.32	.34
11	52.77	.71	.76	.73	.74
12	67.83 (blend)	(.53, .74, .93)	(.53, .73, .96)	.82	.82
13	71.77	.73	.74	.79	.76
14	72.59	.56	.58	.58	.58
15	87.99	.99	8.02	.96	.99
16	92.06 (blend)	(1.88, .14)	(1.88, .14)	.10	.08
17	908.21	.17	.15	.24	.18
18	18.65	.62	.61	………	.63
19	35.85 (poor)	.90	.90	………	.88
20	48.25	.22	.25	.27	.25
21	51.83	.77	.80	………	.80
22	61.01 (poor)	.06	(0.89, .12)	………	………
23	71.91	.89	.92	.90	.90
24	91.71	.68	.71	………	.70
25	92.60 (blend)	(.45, .70)	(.46, .71)	.64	.62
26	1,007.55	.54	.56	.48	.55
27	11.20	.20	.22	.24	.22
28	27.04	.04	.04	6.92	.04
29	32.14	.13	.13	.12	.13
30	46.41	.40	.42	.27	.41
31	53.14	.13	.14	.14	.14
32	65.58	.57	.55	.46	.57
33	70.60	.59	.59	.53	.59
34	74.17	.14	.17	.13	.15
35	84.61	.60	.62	.56	.61
36	89.38	.42	.39	.31	.40
37	95.15	.15	.16	.15	.15
38	1,103.44	.46	.42	.44	.44
39	10.67	.69	.69	.67	.68
40	16.02	.03	.03	5.97	.03
41	22.15	.14	.13	.10	.14
42	31.85	.86	.89	………	.87
43	36.78	.76	.75	………	.76
44	40.62	.65	.65	.58	.64
45	47.53	.52	.54	………	.53
46	52.86	.86	.87	………	.86
47	58.93	.98	.95	.90	.95
48	77.09	.08	.10	………	.09
49	95.02	.00	4.99	………	5.00
50	1,212.66	.68	.69	………	.68

a Garing, Nielsen, and Rao [24].

b Price and coworkers [33].

c Mizushima et al. [12].

d Numerical average of the 4 determinations. If a number differed by 0.04 cm^−1^ or more from the average of the other 3, it was excluded from the average.

**Table 4:** Substructure of v_4_ band of *CH*_4_ from 1,200 to 1,370 cm^−1^

Serial number	This work *v* cm^−1^ (vac.) observed

1	1,216.26
2	30.12
3	33.47
4	41.01
5	47.81
6	56.60
7	65.52
8	75.20
9	81.59
10	92.61
11	97.63
12	1,311.42
13	22.09
14	27.20
15	32.41
16	41.79
17	46.70
18	53.06
19	66.04

**Table 5:** Absorption lines of v_2_ fundamental of *H*_2_*O* from 1,800 to 2,000 cm^−1^

Line serial number	This work *v* cm^−1^ (vac.) observed	Rao, Ryan, and Nielsen^a^ *v* cm^−1^ (vac.) observed

1	1,312.55	………
2	18.92	………
3	36.68	………
4	54.83	………
5	61.08	………
6	08 62	………
7	75.07	………
8	94.46	………
9	1,404.99	………
10	17.42	………
11	29.94	………
12	47.93	………
13	52.04	………
14	64.92	64.91
15	81.25	………
		………
16	87.29	………
17	98.80	………
18	1,512.30	………
19	28.57	………
20	b(45.09)	………
		………
21	64.93	64.91
22	94.52	94.51
23	96.29	96.27
24	1,601.25	01.23
25	09.46	………
26	16.77	16.72
27	27.88	………
28	37.53	………
29	40.31	………
30	62.82	………
31	71.49	………
32	90.15	………
33	1,704.49	………
34	10.23	………
35	23.49	………
36	56.81	………
37	68.17	………
38	75.64	………
39	84.95	………
40	b(90.94)	………
41	99.60	………
42	1,810.63	………
43	25.24	………
44	47.80	………
45	69.35	………
46	89.59	………
47	95.19	………
48	1,918.05	………
49	42.60	………
50	88.43	………
51	b(2,016.79)	………

a Rao, Ryan, and Nielsen [22].

b These lines are less accurate.

**Table 6:** Absorption lines of *CO* from 2,020 to 2,240 cm^−1^

Line No.	Plyler, Blaine, and Connor a *v* cm^−1^ (vac.) observed	Hank et al.^b^ *v* cm^−1^ (vac.) calculated

*P* 28	2,022.899	.915
27	27.635	.650
26	32.349	.354
25	37.030	.026
24	41.663	.668
23	46.271	.278
22	………………	………
21	55.391	.402
20	59.911	.916
19	………………	………
18	68.851	.849
17	………………	………
16	77.650	.652
15	82.009	.005
14	86.322	.324
13	90.603	.611
12	94.870	.865
11	99.096	.085
10	2,103.265	.272
9	07.413	.426
8	11.555	.546
7	………………	………
6	19.677	.684
5	23.700	.702
4	………………	………
3	31.639	.635
2	35.554	.549
1	39.432	.429
*R*0	………………	………
1	………………	………
2	2,154.596	.598
3	58.309	.302
4	………………	………
5	65.602	.604
6	………………	………
7	72.759	.761
8	76.287	.286
9	79.761	.774
10	83.226	.226
11	86.636	.641
12	90.010	.020
13	93.357	.361
14	96.661	.665
15	99.929	.933
16	2,203.147	.163
17	06.345	.355
18	09.498	.510
19	12.600	.626
20	15.685	.705
21	18.733	.746
22	21.732 c21.750	.749
23	24.694 24.712	.713
24	27.638	………
25	30.526	………
26	33.362	………
27	36.186	………
28	38.958	………

a Plyler, Blaine, and Connor [13].

b Rank et al. [14].

c New measurements agreeing more closely with calculated values.

**Table 7–A:** Absorption lines of v_3_ fundamental of C^13^O_2_^16^ from 2,240 to 2,300 cm^−1^

Line number	This work *v* cm^−1^ (vac.) observed	Previous work Plyler, Blaine, and Tidwell^a^ *v* cm^−1^ (vac.) observed	Mizushima et al.^b^ cm^−1^ (vac.) observed

*P* 40	2,247.68	2,247.66	………
38	49.68	.68	………
36	51.70	.67	………
34	53.66	………	………
32	55.59	………	………
			………
30	57.52	.52	2,257.48
28	59.41	.42	.40
26	61.29	.30	.35
24	63.13	.14	.30
22	64.95	.96	5.04
			
20	66.75	.76	.85
18	68.54	.55	.65
16	70.29	.31	.37
14	72.03	………	………
12	73.73	.74	.73
10	75.42	………	………
8	77.09	.08	.03
6	78.71	.71	.64
4	80.33	.31	.18
2	81.94	………	………

Line number	This work *v v* cm^−1^ (vac.) observed	Previous work this laboratory c *v* cm^−1^ (vac.) observed	Mizushima et al.^b^ *v* cm^−1^ (vac.) observed

*R* 0	2,284.24	………	………
2	85.79	.76	.83
4	87.31	.33	.34
6	88.81	.80	.83
8	90.23	.20	.29
10	91.62	.62	.73
12	93.10	.12	………
14	94.45	.46	………
16	95.89	………	.91
18	97.18	.20	.26
20	98.50	………	.57
22	99.76	.74	
24	2,301.05	.06	

a Plyler, Blaine, and Tidwell [26].

b Mizushima et al. [12].

c E. K. Plyler and L. R. Blaine, unpublished work.

**Table 7–B:** Absorption lines of v_3_ fundamental of C^12^O_2_^16^ from 2,280 cm^−1^ to 2,390 cm^−1^

Line number	This work *v* cm^−1^ (vac.) observed	Previous work this laboratory^a^ *v* cm^−1^ (vac.) observed	Line number	This work *v* cm^−1^ (vac.) observed	Previous work this laboratory^a^ *v* cm^−1^ (vac.) observed

*P* 68	2.282.21	………	*R* 48	………	2,379.80
66	………	………	50	………	80.74
64	86.93	………	52	………	81.65
62	89.23	………	54	………	82.51
60	………	………	56	2,383.39	83.38
58	93.81	2,293.81	58	84.22	84.21
56	96.03	96.03	60	85.01	85.01
54	98.30	………	62	85.80	85.79
52	2,300.50	………	64	86.55	86.55
50	………	2,302.70	66	………	87.26
48	………	04.84	68	………	87.97
46	………	06.96	70	………	88.64
	………		72	………	89.32
	………		74	………	89.91
*R* 30	………	2,370.36	76	………	90.49
32	………	71.48		………	
34	………	72.65	78	………	91.10
36	………	73.71	80	………	91.61
38	………	74.79	82	………	92.16
	………		84	………	
40	………	75.83	86	………	93.09
42	………	76.87		………	………
44	………	77.88	88	………	93.55
46	………	78.86	90	………	93.96

Calculated values for lines near band center					

*P* 44	………	2,309.05	*R* 0	………	2,349.97
42	………	11.13	2	………	51.50
40	………	13.19	4	………	53.01
38	………	15.22	6	………	54.49
36	………	17.23	8	………	55.95
34	………	19.21	10	………	57.38
32	………	21.17	12	………	58.78
30	………	23.11	14	………	60.16
28	………	25.02	16	………	61.52
26	………	26.91	18	………	62.86
24	………	28.77	20	………	64.16
22	………	30.60	22	………	65.44
20	………	32.41	24	………	66.70
18	………	34.20	26	………	67.93
16	………	35.97	28	………	69.13
14	………	37.71			
12	………	39.43			
10	………	41.12			
8	………	42.78			
6	………	44.42			
4	………	46.04			
2	………	47.63			

a E. K. Plyler and L. R. Blaine, unpublished work.

**Table 8:** Absorption lines of *HBr*^79^ and *HBr*^81^ together with single unresolved peaks at lower resolution (2,390–2,750 cm^−1^)

Serial number	This work high resolution isotopic components *v* cm^−1^ (vac.) observed	High resolution average	This work low resolution single peak *v* cm^−1^ (vac.) observed

Br^81^	2,392.61	} 2,392.78	………
1			
Br^79^	92.94		
Br^81^	2,412.73	} 2,412.89	2,412.93
2			
Br^79^	13.06		
Br^81^	32.43	} 32.60	32.65
3			
Br^79^	32.77		
Br^81^	51.73	} 51.91	51.94
4			
Br^79^	52.08		
Br^81^	70.61	} 70.79	70.82
5			
Br^79^	70.97		
Br^81^	89.08	} 89.26	89.30
6			
Br^79^	89.43		
Br^81^	2,507.11	} 2,507.30	2,507.24
7			
Br^79^	07.48		
Br^81^	24.71	} 24.90	24.83
8			
Br^79^	25.08		
Br^81^	41.87	} 42.06	41.99
9			
Br^79^	42.25		
Br^81^	74.80	} 74.99	74.98
10			
Br^79^	75.19		
Br^81^	90.56	} 90.76	90.67
11			
Br^79^	90.95		
Br^81^	2,605.82	} 2,606.02	2,605.95
12			
Br^79^	06.22		
Br^81^	20.63	} 20.83	20.81
13			
Br^79^	21.03		
Br^81^	34.92	} 35.13	35.03
14			
Br^79^	35.33		
Br^81^	48.71	} 48.92	48.85
15			
Br^79^	49.13		
Br^81^	61.99	} 62.19	62.13
16			
Br^79^	62.39		
Br^81^	74.76	} 74.97	………
17			
Br^79^	75.19		
Br^81^	87.00	} 87.20	………
18			
Br^79^	87.41		
Br^81^	98.68	} 98.90	………
19			
Br^79^	99.11		
Br^81^	2,709.86	} 2,710.07	………
20			
Br^79^	10.29		
Br^81^	20.48	} 20.68	………
21			
Br^79^	20.89		
Br^81^	30.51	} 30.73	………
22			
Br^79^	30.95		
Br^81^	40.02	} 40.24	………
23			
Br^79^	40.46		
Br^81^	48.93	} 49.16	………
24			
Br^79^	49.39		

**Table 9:** Absorption lines of *HCl* from 2,650 to 3,050

Serial number	This work high resolution *v* cm^−1^ (vac.) observed	This work high resolution *v* cm^−1^ (vac.) calculated	This work low resolution *v* cm^−1^ (vac.) observed	M., T., and W.^a^ *v* cm^−1^ (vac.) observed

HCl^35^				

0	2,651.98	2,651.98	………	.94
1	77.74	.75	.86	.77
2	2,703.02	2,703.02	.03	2.95
3	27.79	.79	.76	.75
4	52.05	.05	.15	.01
5	75.77	.77	.85	.77
6	98.95	.95	.96	9.00
7	2,821.58	2,821.57	.56	.59
8	43.63	.63	.60	.63
9	65.10	.10	.14	.14
10	2,906.25	2,906.25	.24	.25
11	25.91	.90	.89	.92
12	44.92	.92	………	.99
13	63.30	.30	.20	.35
14	81.02	.02	………	.05
15	98.07	.07	………	.05
16	3,014.44	3,014.44	………	.50
17	30.10	.11	.12	.12
18	45.07	.08	.02	.15

HCl^37^				

0	2,650.23	2,650.24	………	.17
1	75.96	.96	………	.98
2	2,701.20	2,701.19	.28	.15
3	25.93	.93	.95	.90
4	50.14	.14	………	.13
5	73.84	.83	.89	.82
6	96.98	.98	………	7.01
7	2,819.57	2,819.57	………	.56
8	41.59	.59	………	.56
9	63.02	.03	………	.06
10	2,904.12	2,904.12	.14	.07
11	23.74	.74	.79	.74
12	42.74	.73	.66	.79
13	61.08	.08	.06	.13
14	78.77	.77	………	.80
15	95.79	.79	………	.78
16	3,012.15	3,012.14	.14	.23
17	27.80	.79	.72	.84
18	42.74	.74	.68	.80

a Mills, Thompson and Williams[28].

**Table 10:** Absorption lines of methane from 2,900 to 3,170 cm^−1^

Line number	Plyler, Blaine, and Nowak^a^ *v* cm^−1^ (vac.) observed

*P* 12	b2.895.18
11	b2,906.72
10	b16.36
9	26.86
8	37.34
7	47.92
6	58.20
5	68.67
4	79.00
3	88.27
2	99.10
*R* 0	3,028.84
1	38.58
2	48.25
3	57.79
4	67.30
5	76.74
6	86.02
7	95.22
8	3,104.36
9	13.42
10	22.46
11	31.39
12	40.20
13	48.95
14	57.61
15	66.20

a Plyler, Blaine, and Nowak [11].

b Frequency of strongest component.

**Table 11:** Absorption bands of acetylene (2 bands) from 3,200 to 3,380 cm^−1^

Serial number	This work medium-high resolution *v* cm^−1^ (vac.) observed	This work low resolution *v* cm^−1^ (vac.) observed	C., E., G., and T^b^ medium resolution *v* cm^−1^ (vac.) observed

2	3,219.42 (H_2_O)	………	………
3	25.63	………	.64
4	30.72	………	.72
5	38.31	.44	.29
6	48.19 a48.38	48.41	.34 a48.40
7	48.56	………	.47
8	50.71	.82 (slightly blended)	.65
9	55.59	………	.56
10	60.46	.60 (slightly blended)	.40
11	65.29	.32	.26
12	70.10	………	.04
13	78.22	………	.20
14	83.04	.14	2.93
15	86.63	………	.48
16	95.88	.80	.83
17	3,300.46	………	.35
18	07.27	………	.14
19	17.92	………	.99
20	22.41^a^ 22.59	22.62	22.44
21	22.77	………	………
22	31.29	.31	.18
23	35.59	………	.49
24	39.83	.98	.72
25	44.08	.01 (slightly blended)	3.93
26	48.23	.21	.08
27	55.74 (H_2_O)	………	………
28	65.78 (H_2_O)	.86	………
29	67.67 (H_2_O)	………	………

a Average of two components of nearly equal intensity.

b Christensen, Eaton, Green, and Thompson [23].

**Table 12–A:** Absorption lines of water from 3,400 to 4,000 cm^−1^

Line serial number	High resolution Plyler and Tidwell^a^ *v* cm^−1^ (vac.) observed	Intermediate resolution this work *v* cm^−1^ (vac.) observed

	3,397.20	
1	3,447.20 (7.03)	3,447.21
2	96.63	96.62
3	3,518.97	3,519.05
4	(2 components)	36.44 avg
5	………	57.21
6	70.54	70.48
7	76.89	………
	77.06 avg	77.02 avg
(8)	77.23 3,603.08	3,602.98 (complex)
9	………	38.15
10	59.94	59.94
10–A(CO_2_)	98.00	98.08
11	………	3,701.90
12	3,714.81	3,714.82
13	………	79.38
14	………	3,835.06
15	………	80.52
15–A	3,883.26	83.32
16	3,920.10	3,920.28
17	………	42.86
18	53.11	53.22
19	69.15	69.16
20	90.72	90.74
21	4,008.59	4,008.59
22	44.90	………

a Plyler and Tidwell [29].

**Table 12–B:** Some precisely measured CO_2_ lines of the 021 and 101 bands at 3,609 and 3,716 cm^−1^

Line number	Plyler and Tidwell^a^ *v* cm^−1^ (vac.) observed	Line number	Plyler and Tidwell^a^ *v* cm^−1^ (vac.) observed

021 Band			

*P* 38	3,579.34	*R* 2	(3,615.25)
36	81.31	10	(21.17)
32	85.21	12	22.50
26	90.78	14	23.93
24	92.65	16	25.27
22	94.42	26	31.85
20	(96.22)	40	40.00
16	99.74		
14	3,601.40		
10	04.81		
8	06.48		
6	08.07		

101 Band			

*P* 36	3,682.78	*R* 6	3,720.09
32	86.74	10	22.90
26	92.46	12	24.35
24	94.34	14	(25.72)
20	98.00	18	28.42
18	99.79	20	29.73
14	3,703.29	22	31.01
12	05.02	26	33.48
10	06.70		
8	08.38		

a Plyler and Tidwell [29].

Values in parentheses indicate lines which may be overlapped by water lines.

**Table 13:** Absorption lines of acetylene from 4,040 to 4,130 cm^−1^

Line number	Previous work this laboratory *v* cm^−1^ (vac.) observed

*P* 3	4,084.14
11	64.58
15	54.52
17	49.36
21	38.95
*R* 17	4,130.10
15	26.91
13	22.63
11	18.30
9	13.94
7	09.47
5	04.98
3	4,100.47
1	4,095.84

**Table 14:** Absorption lines of the 2–0 harmonic band of CO from 4,100 to 4,340 cm^−1^

Line number	Plyler, Allen. and Tidwell^a^ *v* cm^−1^ (vac.) observed	Rank et al.^b^ *v* cm^−1^ (vac.) observed

*P* 27	4,132.19	………
26	37.79	.770
25	43.33	………
24	48.817	………
23	54.234	………
22	59.564	………
21	64.852	………
20	70.066	.058
19	75.212	.204
18	80.286	.285
17	85.302	.298
16	90.234	.242
15	95.120	………
14	99.931	………
13	4,204.673	………
12	09.356	………
11	13.962	………
10	18.504	………
9	22.974	954
8	27.371	………
7	31.694	.685
6	35.952	.949
5	40.150	………
4	44.278	.267
3	48.330	………
2	52.308	.307
1	56.226	………
*R* 0	4,263.842	.838
1	67.548	.542
2	71.182	.179
3	74.750	.743
4	78.235	.234
5	81.655	.661
6	85.013	.010
7	88.298	.290
8	91.512	.500
9	94.639	………
10	97.703	………
11	4,300.712	………
12	03.614	………
13	06.482	………
14	09.262	.257
15	11.969	.962
16	14.606	.597
17	17.173	.161
18	19.665	.650
19	22.085	.065
20	24.425	………
21	26.699	………
22	28.878	………
23	31.022	………
24	33.057	.054
25	………	4,335.030
26	………	36.934
27	………	38.763
28	………	40.517
29	………	42.202

a Plyler, Allen, and Tidwell [15].

b Rank et al. [14].

**Table 15:** Absorption lines of N_2_O from 2,520 to 2,580 cm^−1^

*J*	This work *v* cm^−1^ (vac.) observed	Thompson and Williams^a^ *v* cm^−1^ (vac.) obs. laboratory

*P* 45	2,519.01	.05
43	21.27	.29
41	23.50	.52
39	25.71	.72
37	27.87	.88
35	30.03	.03
33	32.15	.17
31	34.25	.27
29	36.31	.35
27	38.37	.41
25	40.38	.42
23	42.38	.42
21	44.34	.38
19	46.27	.32
17	48.19	.23
15	50.07	.13
13	51.92	2.01 (1.97 calc.)
*R* 0	2,564.18	.24
7	69.82	.86
11	72.87	.94
13	74.38	.45
16	76.56	.59
18	77.99	8.04

a Thompson and Williams [31].

**Table 16:** Polystyrene absorption bands

Previous measurement wavelength (air) *μ*	New measurement this work wavelength (air)	This work cm^−1^ (vac.)

3.3026	………	………
3.422	………	………
3.507	………	………
5.138	………	………
5.343	………	………
5.549	………	………
6.238	6.2427	1,601.4_5_
………	6.3151	1,583.0_8_
6.692	………	………
………	8.4615	1,181.5_0_
8.662	8.6626	1,154.0_8_
………	9.3524	1,068.9_6_
9.724	9.7226	1,028.2_6_
11.035	11.028	906.5_0_
………	14.316	698.3_3_

## Footnotes

3 Figures in brackets indicate the literature references at the end of this paper.
